# Outcomes of Single Corticosteroid Injection in De Quervain's Tenosynovitis

**DOI:** 10.7759/cureus.71928

**Published:** 2024-10-20

**Authors:** Asif Afridi, Bilal Ahmad, Hassaan Ahmed

**Affiliations:** 1 Trauma and Orthopedics, Hayatabad Medical Complex Peshawar, Peshawar, PAK; 2 Trauma and Orthopedics, Queen Elizabeth Hospital Birmingham, Birmingham, GBR; 3 Orthopedics, Queen Elizabeth Hospital Birmingham, Birmingham, GBR

**Keywords:** corticosteroid injection, ligament injury wrist, quervain's tenosynovitis, wrist overuse, wrist pain

## Abstract

Objectives: The aim is to demonstrate the effectiveness of steroid injection in treating De Quervain disease. This study took place in the Orthopedic and Spine unit of Hayatabad Medical Complex. This study started in June 2022 and ended in February 2023.

Materials and methods: Seventy-five patients and 76 hands with De Quervain's Tenosynovitis were recruited for the study. Of these 75 patients, 54 (72%) were female and 21 (28%) were male. The diagnosis of De Quervain's Tenosynovitis was established from the patients' histories, physical examinations, and special tests. One ml of local anesthetic (2% of xylocaine) mixed with 1 ml of Corticosteroid (Depo-Medrol®, containing methylprednisolone acetate 40 mg/ml) were injected between abductor pollicis longus (APL) and extensor pollicis brevis (EPB), 3-4 cm proximal to radial styloid on the effected side. Patients were reviewed at two- and three-weeks period after injection.

Results: Sixty-three (84%) patients were completely pain-free, 9 (12%) patients had mild pain, while 2 (2.2%) patients had no improvement in pain status at all. 1 (1.3%) patient didn’t show up on the second follow-up.

Conclusion: Injecting corticosteroids into the first compartment of the dorsal extensor tendon is a good treatment for De Quervain's tenosynovitis. The incidence of De Quervain's disease is higher in females and in the right hand. Only a limited subset of individuals who have not responded to non-invasive and minimally invasive treatments can be considered for surgical intervention. The study's limitations include a small sample size and fewer follow-ups. Further studies, especially randomized controlled trials with large sample sizes and long follow-ups, are needed to determine the long-term efficacy of steroid injection in De Quervain's tenosynovitis and whether the documented effect is likely to have occurred due to the treatment.

## Introduction

The painful and frequently disabling disorder known as De Quervain tenosynovitis affects the wrist’s first dorsal compartment. The abductor pollicis longus (APL) and extensor pollicis brevis (EPB) tendons, which cross under the extensor retinaculum in the first dorsal compartment of the wrist, have a sheath that thickens and accumulates mucopolysaccharide [1]. The fibrous band known as the extensor retinaculum, which is affixed to the underlying radius, stops the extensor tendons from bowstringing off the wrist's dorsum. The Swiss physician de Quervain, who initially documented a case series of five individuals in 1895, is credited with giving the condition its name. It is estimated that the prevalence is 1.3% for women and 0.5% for men [2]. This condition is common in people who do tasks that need them to use their hands and thumbs repeatedly, such as musicians, athletes, office workers, etc. The pathophysiology of De Quervain tenosynovitis is not clear yet. A number of papers in recent years have documented a connection between De Quervain disease, pregnancy, and breastfeeding [3]. Other risk factors for this condition include rheumatoid arthritis, diabetes, past wrist injuries, and female gender. Osteoarthritis of the first carpometacarpal (CMC) joint, ganglia, infectious tenosynovitis, Wartenberg’s syndrome, and intersection syndrome are among the differential diagnosis [4]. The initial line of treatment is non-surgical, and includes splinting, ice, rest, therapeutic exercise, and non-steroidal anti-inflammatory medications [5]. For individuals who do not respond to the aforementioned treatments, corticosteroid injections are the cornerstone of care. Additional treatments that have been described are acupuncture [6], ultrasound-guided percutaneous needle tenotomy and platelet-rich plasma (PRP) injection [7], and prolotherapy [8]. In situations where there is doubt about the diagnosis, imaging modalities, including magnetic resonance imaging and ultrasound, can be helpful [9]. Injections of steroids are a commonly used treatment, and systematic reviews and randomized control trials (RCTs) have demonstrated their effectiveness [10,11].

A study carried out at Brown University School of Medicine America found that injections of corticosteroids alleviated symptoms in 28 out of 42 patients, yielding a success rate of 66% [5]. Using corticosteroids to treat De Quervain disease had a 95% success rate, according to another study out of Thailand's Khon Kaen University [12]. Both non-surgical and surgical options are available for the treatment of De Quervain tenosynovitis. In most cases, non-surgical care is sufficient to alleviate symptoms; specifically, the most successful treatment, according to the available literature, is the injection of corticosteroids, which has an 83% cure rate. The precise mechanism of action of injectable therapy is yet unknown, although it is commonly believed that the anti-inflammatory effects of corticosteroids are responsible for its success.

## Materials and methods

From June 2022 to February 2023, a total of nine months, the research took place in the Orthopedic and Spine Surgery Unit, Hayatabad Medical Complex, Peshawar. The research comprised 75 persons; 54 (72%) were female, and 21 (28%) were male. Forty-four (58.66%) patients had right, 30 (40%) had left hand, and 1 (1.3%) had bilateral hand involvement. Seventy-four patients had unilateral conditions, while one female had a bilateral condition. The mean age of the patients was 51.64±6.81 years (ranging from 40 to 65 years). The mean duration of symptoms was 4.4±1.1 months (ranging from three to seven months). Verbal consent was taken before patients’ enlisting to study. The diagnosis was made from the patient’s history and physical examination (tenderness over the radial styloid of the effected wrist and positive Finkelstein test). X-rays of the effected wrist were taken to rule out other pathologies mimicking De Quervain tenosynovitis and fractures. Ultrasound was used to confirm the diagnosis of De Quervain tenosynovitis.

Inclusion criteria

The inclusion criteria included (1) manual workers, (2) no history of prior wrist/hand surgery and fracture, (3) positive Finkelstein test, and (4) no chronic illness.

Exclusion criteria

The exclusion criteria included (1) traumatic/pathological wrist fractures, (2) previous wrist/hand surgery, and (3) osteoarthritis of the radio carpal or first carpometacarpal joints.

Injection technique

A cocktail of one ml of 2% xylocaine and one ml of methylprednisolone acetate (Depo-Medrol® 40 mg/ml) was prepared in a 3cc syringe. The skin of the hand was prepared with chlorhexidine and alcohol wipes. Under aseptic conditions, the first dorsal compartment of the wrist was identified, and the syringe’s needle was introduced. The cocktail was infused in a distal to proximal direction. The plastic bandage was applied to the needle prick site.

The tendon sheath must be distended by the cocktail injection. Using this approach, the compartment becomes more prominent. It is common to hear or feel a "pop" after injecting into the tendon sheath. Due to the excruciating nature of De Quervain's disease, lidocaine provides nearly immediate symptom relief for many patients.

## Results

**Figure 1:**
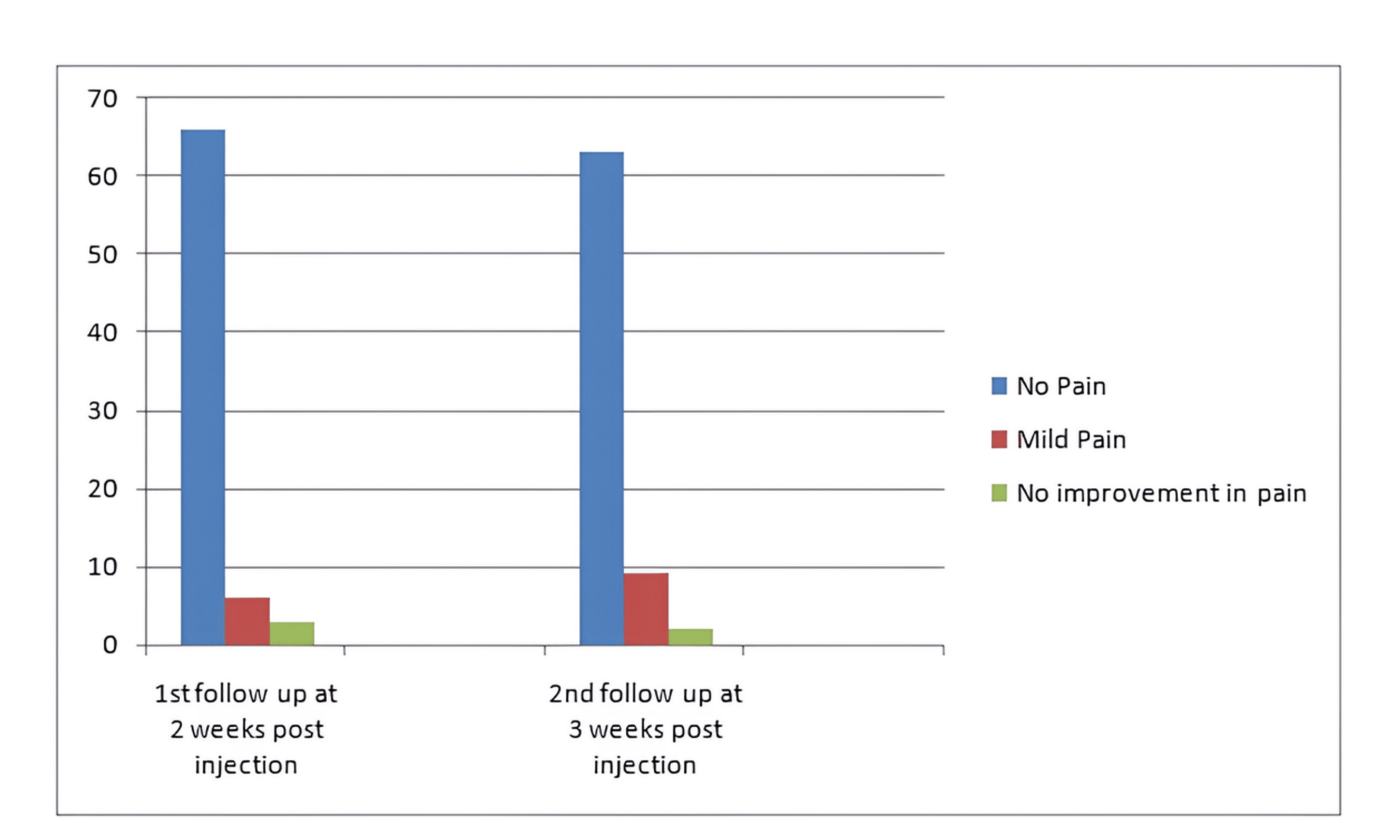
Graphical representation of post-injection pain statistics.

Symptomatic relief from pain was achieved in all patients with a local anesthetic component of the cocktail right after the injection. Patients were followed up during the second- and third-week period post-injection (Figure 1). At the second week post-injection, 66 (88%) of patients had no pain, 6 (8%) had mild pain, which was not limiting their daily routine activities, while 3 (4%) patients had no improvement in pain with injection. On the third week post-injection, 63 (84%) patients were completely pain-free, 9 (12%) patients had mild pain, while 2 (2.2%) patients had no improvement in pain status at all. One (1.3%) patient didn’t show up on the third-week follow-up (Tables 1-3).

**Table 1:** Gender of patients.

Gender	Frequency	Percentage (%)
Female	54	72
Male	21	28

**Table 2:** Laterality of condition.

Laterality	Number (n)	Percentage (%)
Right hand	44	58.6
Left hand	30	40
Both hands	1	1.3

**Table 3:** Average age of the patients and average duration of symptoms.

Mean age	Mean duration of symptoms
51.64±6.81 years (ranging 40-65 years)	4.4±1.1 months (ranging from three to seven months).

## Discussion

De Quervain Tenosynovitis has advanced in terms of diagnosis and treatment since De Quervain's 1895 description of the condition. For the majority of patients with De Quervain tenosynovitis, corticosteroid injections represent the most successful course of treatment. The success rate of corticosteroid injection alone is even higher at 83% [13]. The injection of corticosteroids carries the risk of tendon rupture (APL, EPB), skin depigmentation, infection, superficial radial nerve neuritis, and subcutaneous fat atrophy [14-16]. Corticosteroid injection results could vary from one group of people to another. One study at Brown University's medical school in the US found that injecting patients with corticosteroids relief their symptoms in 28 of 42 cases, with a favorable result of 66% [5]. Another study from Thailand's Khon Kaen University found that injecting corticosteroids into patients with De Quervain tenosynovitis resulted in a 95% success rate [12]. This study found that injecting corticosteroids into the patients with De Quervain Tenosynovitis relieved their symptoms. The bulk of the participants in this research were women. This lines up with the findings of Wolf et al., who demonstrate that the prevalence of De Quervain's disease was substantially greater in women than in men [13]. Overuse syndrome can develop because women are more likely to be involved in domestic tasks that need repetitive motions, such as cleaning and washing clothes and utensils, caring for children, and using technological gadgets. The success rate of locally injected corticosteroids in this study is 84%, which is in lines up with a study done by Richie et al. [17] that showed the success rate of locally injected corticosteroids in De Quervain disease to be 83%. Different trials employ different injection techniques and different corticosteroids. Ultrasound-guided injection is described to be more effective and safer as described by different researchers. In this study, we use single-point injection in which corticosteroid injection is made at a single point along the radial side of the wrist at the most tender point along the path, followed by extensor pollicis brevis and abductor pollicis longus. In our study, methylprednisolone acetate, one ml containing 40 mg of the drug, is used along with one ml of 2% xylocaine. A total of 44 (58.6%) right hands, 30 (40%) left hands, while 1(1.3%) both hands were affected by De Quervain disease in this study. Rossi et al. [18] found that volleyball players are more likely to experience De Quervain's tenosynovitis in their right wrist, which is consistent with this finding.

## Conclusions

Injecting corticosteroids into the first compartment of the dorsal extensor tendon is a good treatment for De Quervain's tenosynovitis. The incidence of De Quervain disease is higher in females and in the right hand. Only a limited subset of individuals who have not responded to non-invasive and minimally invasive treatments can be considered for surgical intervention. The limitation of the study includes a small sample size and fewer number of follow-ups, further studies especially randomized controlled trials are needed with a large sample size and long follow-ups to know the long-term efficacy of steroid injection in De Quervain's tenosynovitis and to determine whether the documented effect is likely to have occurred due to the treatment.
